# Association between Thrombophilic Genes Polymorphisms and Recurrent Pregnancy Loss Susceptibility in the Iranian Population: a Systematic Review and Meta-Analysis

**DOI:** 10.22034/ibj.22.2.78

**Published:** 2018-03

**Authors:** Mahdieh Kamali, Sedigheh Hantoushzadeh, Sedigheh Borna, Hossein Neamatzadeh, Mahta Mazaheri, Mahmood Noori-Shadkam, Fatemeh Haghighi

**Affiliations:** 1 Department of Perinatology, School of Medicine, Tehran University of Medical Sciences, Tehran, Iran; 2 Maternal-Fetal and Neonatal Research Center, Tehran University of Medical Sciences, Tehran, Iran; 3 Department of Medical Genetics, Shahid Sadoughi University of Medical Sciences, Yazd, Iran; 4 Mother and Newborn Health Research Center, Shahid Sadoughi University of Medical Sciences, Yazd, Iran; 5 Department of Gynecology and Obstetrics, Shahid Sadoughi University of Medical Sciences, Yazd, Iran

**Keywords:** Recurrent miscarriage, Thrombophilia, Factor V leiden, Prothrombin, Meta-analysis

## Abstract

Studies have indicated that thrombophilic genes polymorphisms are associated with recurrent pregnancy loss (RPL) in the Iranian population. We aimed to evaluate the precise association between thrombophilic genes polymorphisms (*MTHFR C677T*, *MTHFR A1298C*, *Prothrombin G20210A*, *FVL* *G1691A*, and *PAI-1 4G/5G*) and RPL risk in the Iranian population. PubMed, Web of Science, Google Scholar, and ISC were searched for eligible articles published up to April 1, 2017. In total, 37 case-control studies in 18 relevant publications were selected: 1,199, 1,194, 630, 830, and 955 RPL cases and 1,079, 1079, 594, 794, and 499 controls for *MTHFR* C677T, *MTHFR A1298C*,*Prothrombin G20210A*, *FVL G1691A*, and PAI-1 4G/5G, respectively. The results indicated a significant increased risk of RPL in all genetic models in the population. Also, *Prothrombin* G20210A and *FVL G1691A* as well as *PAI-1 4G/5G* polymorphisms were associated with RPL risk in the Iranian population. Hence, thrombophilic genes polymorphisms are associated with an increased RPL risk in the Iranian population.

## INTRODUCTION

The miscarriage of three or more consecutive pregnancies in the first or early second trimester is termed as recurrent pregnancy loss (RPL)[1]. Several etiological factors, including endocrinologic problems, uterine structural, chromosomal anomalies, and antiphospholipid antibody syndrome can be the causes of some RPL cases. However, in many cases, the pathogenesis of RPL remains unknown[2,3]. For more than two decades, researchers have focused on certain inherited thrombophilic factors that may be the risk of arterial and/or venous thromboses and their possible association with pregnancy complications such as early pregnancy loss[4]. It is estimated that the thrombophilia is a common cause of RPL and is found in 40-50% of cases[5].

Three common inherited thrombophilia markers, namely Factor V Leiden (*FVL*), Prothrombin G20210A (*PT* *G20210A*), and Methylene tetrahydrofolate reductase (*MTHFR*) *C677T* are candidate genes for venous thromboembolism (VTE)[5]. Various hypotheses were proposed to explain the role of the thrombophilic genes polymorphisms such as *MTHFR C677T*, *MTHFR A1298C*, *Prothrombin G20210A*, *FVL G1691A*, and *PAI-1 4G/5G* in RPL[6,7]. A large number of studies have investigated the association between the thrombophilia gene polymorphisms and RPL susceptibility in the Iranian population[8-25]. However, the results were inconsistent or inconclusive, presumably due to the small sample size in these published studies. Undoubtedly, meta-analysis can be used to increase power and answer questions not posed by the individual studies. Therefore, we conducted this systematic review and meta-analysis to investigate the association between the most common polymorphisms of thrombophilic genes (including *MTHFR C677T*, *MTHFR A1298C*, *Prothrombin G20210A*, *FVL G1691A*, and *PAI-1 4G/5G*) and RPL risk in the Iranian population.

## MATERIALS AND METHODS

### Search strategy

To identify eligible studies for this meta-analysis, we searched the PubMed, Web of Science, Google Scholar databases, ISC, and EMBASE. In the search, we considered all eligible articles published up to April 1, 2017 that examined the association between the *MTHFR C677T*, *MTHFR A1298C*, *Prothrombin G20210A*, *FVL G1691A*, and *PAI-1 4G/5G* polymorphisms and RPL risk in the Iranian population. The following key terms were included in our search: “recurrent pregnancy loss”, ‘’recurrent miscarriage’’, ‘’habitual abortion’’, “RPL”, “thrombophilic gene”, “*MTHFR* *C677T*’’, “*MTHFR* A1298C’’, “*Prothrombin* G20210A’’, “*Factor V Leiden G1691A*’’, “*PAI-1 4G/5G*’’, “polymorphism”, “variant”, “gene”, “genotype”, “SNP”, and “allele”. The extracted publications were limited to Persian and English languages and conducted only on human subjects. We retrieved those publications matching the keywords without no restriction, and then the studies were evaluated by reading the title and abstract. We have also screened the reference lists of the retrieved articles for original papers. If there were multiple reports of the same study or overlapping data, only the study with the largest sample sizes or the most recent one was selected in our meta-analysis, and the others were excluded.

### Inclusion and exclusion criteria

The included studies to the meta-analysis had to be consistent with the following criteria: (1) published in full-text, (2) be case-control or cohort design, 3) evaluate the association between *MTHFR C677T*, *MTHFR A1298C*, *Prothrombin G20210A*, *FVL G1691A*, and *PAI-1 4G/5G* polymorphisms and the risk of RPL in the Iranian populations, (4) offered the size of the sample and sufficient data (genotype distributions of both cases and controls were available) for estimating OR with 95% CI, and (5) written in English or Persian. The exclusion criteria were as follows: (1) abstracts, case reports, letter to the editor, and reviews, (2) studies with only case group (no control population), (3) studies on other poly-morphisms of thrombophilic genes, (4) studies without detail genotype frequencies in which calculation of OR is impossible, and (5) duplicate publications of data from the same study.

### Data extraction

Two investigators independently extracted the data using a pre-designed form. Based on the inclusion and exclusion criteria, we extracted the following data from each study: the first author, year of publication, number of RPL patients and controls, genotype and allele frequency, minor allele frequencies (MAFs) in control subjects, and Hardy-Weinberg equilibrium (HWE) test in control subjects. For conflicting evaluation, these two investigators carried out discussions until a consensus was reached.

### Statistical analysis

All the statistical analyses were performed by comprehensive meta-analysis (CMA) version 2.0 software (Biostat, USA). All *P* values were two-tailed with a significant level at 0.05. The strength of associations was assessed by using ORs and 95% CIs, and the significance of pooled ORs was examined by Z_test_. We performed a meta-analysis of the association between *MTHFR C677T* polymorphism and RPL under the allelic model (T vs. C), the homozygote model (TT vs. CC), the heterozygote model (CT vs. CC), the dominant model (TT + CT vs. CC), and the recessive model (TT vs. CT + CC). The *MTHFR A1298C* polymorphism was evaluated using the allelic model (C vs. A), the heterozygote model (AC vs. AA), the homozygote model (CC vs. AA), the dominant model (CC + AC vs. AA), and the recessive model (CC vs. AC + AA). The *Prothrombin G20210A* and *FVL G1619A* polymorphisms were assessed under the allelic model (A vs. G), heterozygote model (GA vs. GG), the homozygote model (AA vs. GG), the dominant model (AA + AG vs. GG), and the recessive model (AA vs. AG + GG). *PAI-1 4G/5G* polymorphism was assessed under the allelic model (4G vs. 5G), the heterozygote model (4G/5G vs. 5G/5G), the homozygote (4G/4G vs. 5G/5G), the dominant model (4G/4G + 4G/5G vs. 5G/5G), and the recessive model (4G/4G vs. 4G/5G + 5G/5G). Heterogeneity assumption was checked by a chi-square-based Q-test, and I^2^ statistics was calculated to quantify the proportion of the total variation across studies due to heterogeneity[26]. The heterogeneity was considered significant if either the Q statistics had *p* < 0.1 or I^2^ > 50%. An I^2^ value of 0% represents no heterogeneity, and with the values of 25%, 50%, 75%, or more, it represents low, moderate, high, and extreme heterogeneity, respectively. A *P* value greater than 0.10 indicated the lack of heterogeneity among studies; therefore, the fixed-effects model (Mantel-Haenszel method) was used to calculate pooled OR[27]. Otherwise, the fixed-effects model (Mantel-Haenszel approach) was used. HWEs were calculated with goodness-of-fit tests (i.e., chi-square or Fisher’s exact tests). A value of *p* < 0.01 signified a departure from HWE[28]. One-way sensitivity analyses were carried out by consecutively omitting one study at a time to assess the power of the meta-analysis findings[29]. Visual inspection of the asymmetry of funnel plots was carried out to assess potential publication bias. Begg’s funnel plot, a scatter plot of effect against a measure of study size, was generated as a visual aid to detect bias or systematic heterogeneity[30]. Publication bias was assessed by Egger’s test; *p* < 0.05 was considered statistically significant[31]. Sensitivity analysis was performed to evaluate the stability of the results by removing the studies, but not in HWE.

## RESULTS

### Characteristics of included studies

Based on the search criteria, 53 individual literatures were found. After screening the titles and abstracts, 35 publications that did not meet the criteria were excluded. These studies were reviews, short reports, case reports, and other polymorphisms of *MTHFR*, *Prothrombin*, *FVL*, and *PAI*-1 genes. As summarized in Tables 1 and 2, a total of 37 case-control studies in 18 publications[8-25] were selected in the final meta-analysis, including 1,199 RPL cases and 1,079 controls for *MTHFR C677T* (from ten studies), 1,194 RPL cases and 1079 controls for *MTHFR A1298C* (from ten studies), 630 RPL cases and 594 controls for *Prothrombin G20210A* (from five studies), 830 RPL cases and 794 controls for *FVL G1691A* (from seven studies), and 955 RPL cases and 499 controls for *PAI-1 4G/5G* (from five studies). Genotype distributions in the controls of ten case-control studies were not in agreement with HWE.

**Table 1:** Characteristics of studies included in *MTHFR C677T* and *A1298AC* polymorphisms and RPL

First author	Year	Case/Control	Cases					Controls					MAFs	HWE
	
			Genotype			Allele		Genotype			Allele					
**MTHFR C677T**			**CC**	**TC**	**TT**	**C**	**T**	**CC**	**TC**	**TT**	**C**	**T**		

Bagheri[8]	2010	61/53	34	22	5	90	32	27	21	5	75	31	0.292	0.756
Jeddi-Tehrani[9]	2011	100/100	43	42	15	128	72	66	25	9	157	43	0.215	<0.001
Kazerooni[10]	2012	60/62	50	6	4	106	14	54	6	2	114	10	0.080	0.006
Poursadegh Zonouzi[11]	2012	89/50	53	30	6	136	42	27	22	1	76	24	0.240	0.144
Idali[12]	2012	106/100	61	36	9	158	54	66	25	9	157	43	0.215	0.009
Eskandari[13]	2013	105/98	43	48	14	134	76	61	30	7	152	44	0.224	0.231
Khaleghparast[14]	2014	30/10	13	17	0	43	17	5	5	0	15	5	0.250	0.291
Yousefian[15]	2014	204/116	96	90	18	282	126	63	43	10	169	63	0.271	0.497
Farahmand[16]	2015	330/350	180	114	36	474	186	230	85	35	545	155	0.221	<0.001
Najafian[17]	2016	114/140	30	48	36	108	120	58	56	0	172	56	0.245	<0.001

**MTHFR 1298C**			**AA**	**CA**	**CC**	**A**	**C**	**AA**	**CA**	**CC**	**A**	**C**		

Bagheri[8]	2010	61/53	24	28	9	76	46	21	24	8	66	40	0.377	0.791
Jeddi-Tehrani[9]	2011	100/100	69	27	4	165	35	94	6	0	194	6	0.030	0.757
Poursadegh Zonouzi[11]	2012	89/50	35	46	8	116	62	13	34	3	60	40	0.400	0.003
Idali[12]	2012	106/100	40	46	20	126	86	94	6	0	194	6	0.030	0.757
Sheikhha[18]	2012	60/60	8	45	7	51	49	34	26	0	94	26	0.216	0.032
Khaleghparast[19]	2014	30/10	11	13	6	35	25	5	2	3	12	8	0.040	0.065
Yousefian[15]	2014	204/116	98	81	25	277	131	68	39	9	175	57	0.245	0.316
Farahmand[16]	2015	330/350	134	152	44	420	240	329	20	1	678	22	0.031	0.250
Arabkhazaeli[19]	2016	100/100	100	0	0	200	0	100	0	0	200	0	0.00	0.250
Najafian[17]	2016	114/140	30	48	36	108	120	58	56	0	172	56	0.245	<0.001

MAFs, minor allele frequencies; HWE, Hardy-Weinberg equilibrium

**Table 2:** Characteristics of studies included in *Prothrombin* G20210A, *FVL G1691A*, and *PAI-1 4G/5G* polymorphisms and RPL

First author	Year	Case/control	Cases					Controls					MAFs	HWE
	
			Genotype			Allele		Genotype			Allele			
**Prothrombin G20210A**			**GG**	**AG**	**AA**	**G**	**A**	**GG**	**AG**	**AA**	**G**	**A**		

Bagheri[8]	2011	70/60	48	22	0	118	22	57	3	0	117	3	0.025	0.842
Kazerooni[10]	2013	60/60	52	8	0	112	8	54	6	0	114	6	0.050	0.683
Teremmahi Ardestani[21]	2013	80/80	80	0	0	160	0	80	0	0	160	0	0.00	0.683
Parand[21]	2013	90/44	88	2	0	178	2	43	1	0	87	1	0.011	0.939
Farahmand [16]	2015	330/350	316	14	0	646	14	340	10	0	690	10	0.014	0.786

**FVL 1619 G/A**			**GG**	**AG**	**AA**	**G**	**A**	**GG**	**AG**	**AA**	**G**	**A**		

Bagheri[8]	2011	70/60	70	0	0	140	0	60	0	0	120	0	0.00	0.786
Kazerooni[10]	2013	60/60	43	12	5	98	22	54	4	2	112	8	0.066	0.003
Torabi[22]	2012	100/100	87	12	1	186	14	96	4	0	196	4	0.002	0.833
Teremmahi Ardestani[20]	2013	80/80	78	2	0	158	2	79	1	0	159	1	0.006	0.955
Parand[21]	2013	90/44	72	15	3	159	21	38	6	0	82	6	0.068	0.627
Farahmand[16]	2015	330/350	302	28	0	632	28	340	10	0	690	10	0.014	0.786
Arabkhazaeli[19]	2016	100/100	95	5	0	195	5	91	9	0	191	9	0.045	0.637

**PAI-1 4G/5G**			**5G5G**	**5G4G**	**4G4G**	**5G**	**4G**	**5G5G**	**5G4G**	**4G4G**	**5G**	**4G**		

Jeddi-Tehrani[9]	2011	100/100	60	31	9	151	49	72	27	1	171	29	0.145	0.373
Aarabi[23]	2010	54/99	21	23	10	65	43	31	66	2	128	70	0.353	<0.001
Idali[12]	2012	106/100	35	53	18	123	89	72	27	1	171	29	0.145	0.373
Khosravi[24]	2013	595/100	128	208	85	464	378	72	27	1	171	29	0.145	0.373
Shakarami[25]	2015	100/100	33	50	17	116	84	45	50	5	140	60	0.300	0.056

MAFs, minor allele frequencies; HWE, Hardy-Weinberg equilibrium

### Quantitative synthesis

**Fig. 1:**
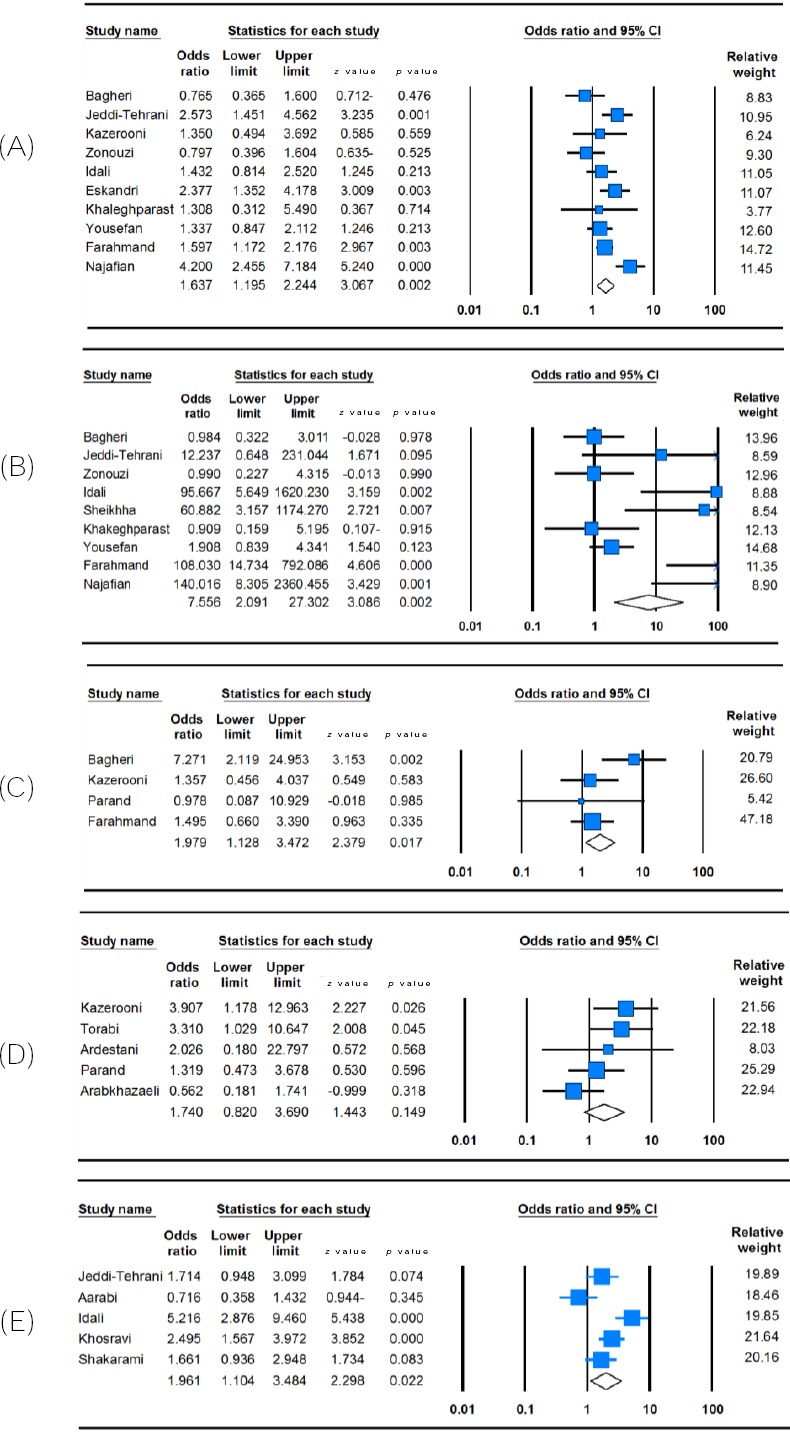
Forest plot of RPL susceptibility associated with thrombophilic genes polymorphisms. (A) *MTHFR C677T* (dominant model: TT + CT vs. CC); (B) *MTHFR A1298C* (homozygote model; AA vs. CC); (C) *Prothrombin* *G20210A* (allele model; A vs. G); (D) *FVL G1619A* (heterozygote model: GA vs. GG), and (E) *PAI-1 4G/5G* (dominant model: 4G/4G + 4G/5G vs. 5G/5G). For each study, the estimation of OR and its 95% CI are plotted with a square and a horizontal line. A diamond indicates the pooled OR with 95% CI.

Table 3 listed the main results of the meta-analysis of *MTHFR* C677T and *A1298C* polymorphisms and RPL risk in the Iranian population. When all the eligible studies were pooled into the meta-analysis of *MTHFR* C677T polymorphism, a significant increased risk of RPL was observed in the allelic model (T vs. C: OR = 1.700, 95% CI = 1.208-2.393, *p* = 0.002), the heterozygote model (CT vs. CC: OR = 1.670, 95% CI = 1.215-2.295, *p* = 0.002), the homozygote model (TT vs. CC: OR = 2.409, 95% CI = 1.291-4.497, *p* = 0.006), the dominant model (TT + CT vs. CC: OR = 1.847, 95% CI = 1.264-2.699, *p* = 0.002, Fig. 1A), and the recessive model (TT vs. CT + CC: OR = 1.858, 95% CI = 1.087-3.177, *p* = 0.024). In addition, when all the eligible studies were pooled into the meta-analysis of *MTHFR A1298C* polymorphism, a significant association was observed in the allelic model (C vs. A: OR = 3.190, 95% CI = 1.467-6.936, *p* = 0.003), the heterozygote model (AC vs. AA: OR = 0.344, 95% CI = 1.344-8.321, *p* = 0.009), the homozygote model (CC vs. AA: OR = 5.073, 95% CI = 1.710-15.051, *p* = 0.003, Fig. 1B), the dominant model (CC + AC vs. AA: OR = 4.006, 95% CI = 1.578-10.169, *p* = 0.003), and the recessive model (CC vs. AC + AA: OR = 5.061, 95% CI = 1.668-15.361, *p* = 0.004).

**Table 3:** The meta-analysis of thrombophilic genes polymorphisms and RPL risk

Polymorphism	Study Number	Genetic Model	Type of Model	Heterogeneity		Odds Ratio				Publication Bias	
		
				I^2^ (%)	P_H_	OR	95% CI	Z_test_	P_OR_	P_Beggs_	P_Eggers_
MTHFR C677T											
	11	T vs. C	Random	83.71	<0.001	1.700	1.208-2.393	3.044	0.002	0.533	0.854
	11	TC vs. CC	Random	64.10	0.002	1.670	1.215-2.295	3.163	0.002	0.275	0.459
	10	TT vs. CC	Random	69.40	0.001	2.409	1.291-4.497	2.761	0.006	0.107	0.132
	11	TT + TC vs. CC	Random	78.55	<0.001	1.847	1.264-2.699	3.171	0.002	0.640	0.743
	10	TT vs. TC + CC	Random	60.50	0.007	1.858	1.087-3.177	2.264	0.024	0.020	0.056
MTHFR A1298C											
	9	C vs. A	Random	94.60	<0.001	3.190	1.467-6.936	2.928	0.003	0.754	0.752
	9	CA vs. AA	Random	92.68	<0.001	3.344	1.344-8.321	2.595	0.009	0.602	0.995
	9	CC vs. AA	Random	70.70	<0.001	5.073	1.710-15.051	2.927	0.003	0.076	0.015
	9	CC + CA vs. AA	Random	93.67	<0.001	4.006	1.578-10.169	2.920	0.003	0.916	0.937
	9	CC vs. CA + AA	Random	74.31	<0.001	5.061	1.668-15.361	2.863	0.004	0.348	0.022
Prothrombin G20210A											
	4	A vs. G	Fixed	45.61	0.138	1.979	1.128-3.472	2.379	0.017	0.308	0.902
	4	AA + AG vs. GG	Fixed	52.94	0.095	2.060	1.162-3.652	2.474	0.013	0.308	0.881
FVL 1619 G/A											
	6	A vs. G	Fixed	41.08	0.131	2.252	1.504-3.373	3.942	<0.001	0.452	0.572
	5	AG vs. GG	Fixed	43.13	0.134	1.695	0.980-2.932	1.888	0.059	0.462	0.786
	3	AA vs. GG	Fixed	0.00	0.995	3.277	0.861-12.473	1.740	0.082	1.000	0.425
	6	AA + AG vs. GG	Fixed	44.02	0.112	2.217	1.447-3.395	3.658	<0.001	0.452	0.626
	3	AA vs. AG + GG	Fixed	0.00	0.985	2.867	0.756-10.868	1.549	0.121	1.000	0.333
PAI-1 4G/5G											
	5	4G vs. 5G	Random	75.26	0.003	2.159	1.427-3.244	3.244	<0.001	0.806	0.925
	5	4G/5G vs. 5G/5G	Random	86.95	<0.001	1.799	0.852-3.796	1.541	0.123	0.220	0.087
	5	4G/4G vs. 5G/5G	Fixed	32.94	0.202	9.811	4.782-20.130	6.227	<0.001	0.462	0.071
	5	4G/4G + 4G/5G vs. 5G/5G	Random	79.84	0.001	1.961	1.104-3.484	2.298	0.022	0.806	0.427
	5	4G/4G vs. 4G/5G + 5G/5G	Fixed	22.62	0.270	10.161	4.975-20.754	6.363	<0.001	0.462	0.050

Table 3 summarizes the ORs with corresponding 95% CIs for association of *Prothrombin G20210A*, *FVL G1691A*, and *PAI-1 4G/5G* polymorphisms with RPL risk in the Iranian population. Among the eligible studies were pooled to the meta-analysis of *Prothrombin G20210A* polymorphism, only allelic and dominant model was applicable because they provided the genotypes of AA + GA vs. GG. Therefore, the pooled analyses showed a significant association between *Prothrombin G20210A* polymorphism and RPL in the Iranian population in the allelic model (A vs. G: OR = 1.979, 95% CI = 1.128-3.472, *p* = 0.017, Fig. 1C) and the dominant model (AA + GA vs. GG: OR = 2.060, 95% CI = 1.162-3.652, *p* = 0.013). When all the eligible studies were pooled into the meta-analysis of *FVL G1691A* polymorphism, we observed a significant increased risk of RPL under allelic model (A vs. G: OR = 2.252, 95% CI = 1.504-3.373, *p* < 0.001, Fig. 1D) and dominant model (AA+GA vs. GG: OR = 2.217, 95% CI = 1.447-3.395, *p* < 0.001), but not in the heterozygote model (AG vs. GG: OR = 1.695, 95% CI = 0.980-2.932, *p* = 0.059), the homozygote (AA vs. GG: OR = 3.277, 95% CI = 0.861-12.473, *p* = 0.082), and the recessive model (AA vs. AG + GG: OR=2.867, 95% CI = 0.756-10.868, *p* = 0.121). In addition, there was a significant association between *PAI-1 4G/5G* polymorphism and RPL in the

Iranian population in the allelic model (4G vs. 5G: OR = 2.159, 95% CI = 1.427-3.244, *p* < 0.001), the homozygote model (4G/4G vs. 5G/5G: OR = 9.811, 95% CI = 4.782-20.130, *p* < 0.001), the dominant model (4G/4G + 4G/5G vs. 5G/5G: OR = 1.961, 95% CI = 1.104-3.484, *p* = 0.022, Fig. 1E), and the recessive model (4G/4G vs. 4G/5G + 5G/5G: OR = 10.161, 95% CI = 4.975-20.754, *p* < 0.001), but not in the heterozygote model (4G/5G vs. 5G/5G: OR = 1.799, 95% CI = 0.852-3.796, *p* = 0.123).

### Sensitivity analysis

To evaluate the influence of individual studies on the risk of RPL, the studies were sequentially deleted from this meta-analysis, and the pooled ORs were performed. However, the results did not change exactly, which verify that no individual studies significantly affected the pooled ORs. Additionally, sensitivity analysis was performed after excluding HWE-violating studies, and the corresponding pooled ORs were not materially altered, indicating that our results are statistically robust (not shown).

### Publication Bias

**Fig. 2:**
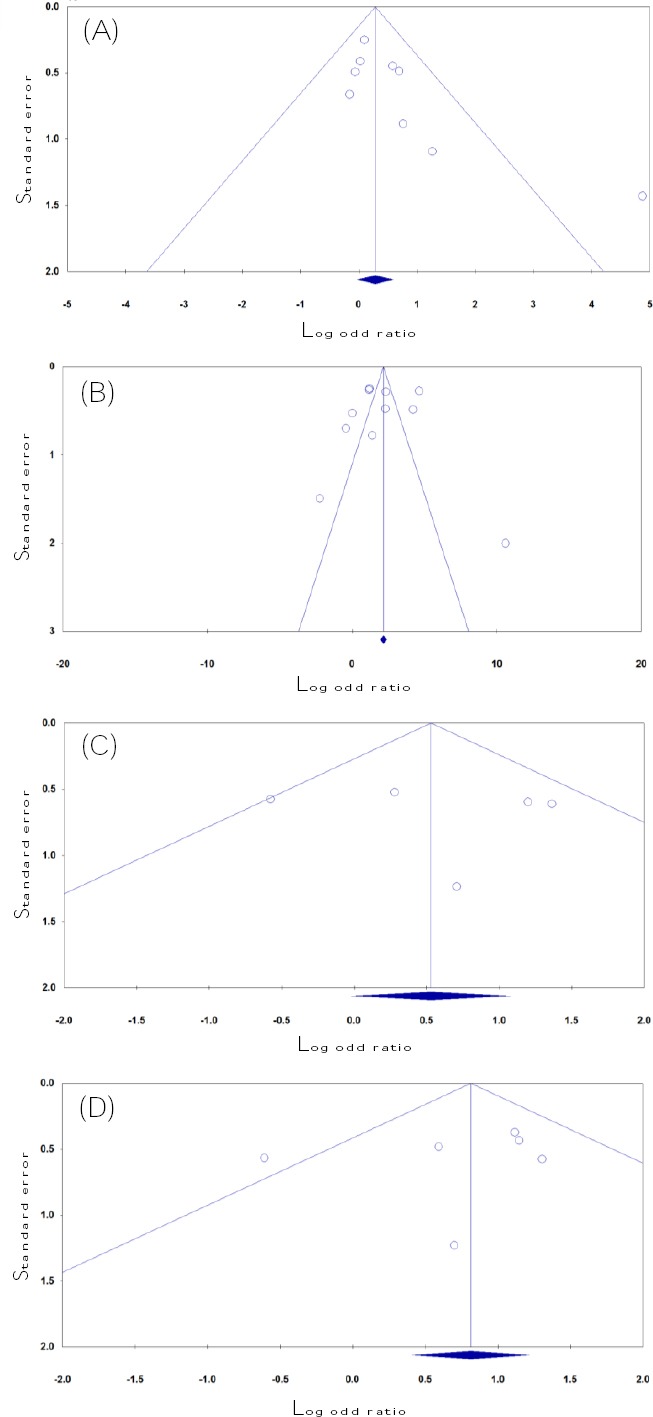
Begg’s funnel plots for thrombophilia gene polymorphisms and RPL risk in the Iranian patients to test the publication bias. (A) *MTHFR C677T* (recessive model: TT vs. CT + CC); (B) *MTHFR A1298C* (dominant model: AA vs. AC + CC); (C) *Prothrombin* G20210A (dominant model: AA + GA vs. GG); (D) *FVL G1691A* (allele model: A vs. G). Each point represents a separate study for the indicated association.

In this meta-analysis, Begg’s funnel plot and Egger’s test were used to assess the publication bias of included studies. The funnel plot revealed no obvious publication bias for *MTHFR* A1298C, *Prothrombin* G20210A, *FVL G1691A*, and *PAI-1 4G/5G*, and this result was confirmed by Begg’s test and Egger’s test (Fig. 2). However, the shapes of the funnel plots revealed obvious asymmetry for *MTHFR* *C677T* and *MTHFR A1298C* in the recessive model (Fig. 2A), suggesting that there were obvious publication biases in these two genetic models. Moreover, the results of Egger’s regression test provided sufficient evidence for publication bias for *MTHFR A1298C* in the recessive model (P_Begg’s_ = 0.348, P_Egger’s_ = 0.022), but not for *MTHFR* *C677T (P*_Begg’s_ = 0.020, P_Egger’s_ = 0.056*)*. Therefore, we used the Duval and Tweedie non-parametric “trim-and-fill” method to adjust the results of publication bias for *MTHFR A1298C* polymorphism recessive model. However, the meta-analysis with and without ‘‘trim-and-fill’’ method did not show different results, showing that the results of this meta-analysis are statistically robust.

## DISCUSSION

It is known that folate is required for the proper development of fetus and placenta[32]. Aberrations in folate pathway such as maternal folate deficiency, maternal hyperhomocysteinemia, and either *MTHFR C677T* or *A1298C* polymorphisms were found to contribute to the etiology of RPL in different populations[33]. Although *MTHFR A1298C* poly-morphism may not responsible for increased total homocysteine, it seems that this polymorphism contributes significantly to the increased homocysteine levels[34,35]. Previous studies have reported that *MTHFR C677T* polymorphism is significantly associated with the increased risk of RPL. For instance, Chen *et al*.[36] in a meta-analysis of 16 articles involving 1420 cases with RPL and 1408 controls reported that *MTHFR C677T* was significantly associated with RPL risk in the Chinese population under all genetics models. Similarly, Wu *et al*.[37] and Cao *et al*.[38] findings supported that the idea that *MTHFR C677T* polymorphism was associated with the increased risk of RPL among Asians, but not Caucasians. Based on these studies, the *MTHFR 677TT* polymorphism has a significant increased likelihood of RPL in Asians, which is in agreement with our conclusion. The data of a meta-analysis by Nair *et al*.[39] showed that *MTHFR A1298C* polymorphism was a genetic risk factor for RPL. However, Cao *et al*.[38] did not find any significant association between *MTHFR A1298C* polymorphism and RPL susceptibility. The combined data, based on previous studies, showed that both *MTHFR C677T* and *MTHFR A1298C* polymorphisms might be a risk factor for RPL.

As for the other two polymorphisms, we also find a significant association of polymorphisms in *Prothrombin G20210A*, *FVL G1691A*, and *PAI-1 4G/5G* with the risk of RPL, which were consistent with the majority but not all previous studies[40-42]. In a meta-analysis conducted by Gao *et al*.[41], the *Prothrombin G20210A* variant was reported to increase the risk of RPL (fetal loss, primary RPL, or secondary RPL)[43], particularly in Europeans and women older than 29 years. In another meta-analysis, Kovalevsky *et al*.[44] found that *FVL G1691A* or *Prothrombin* gene polymorphisms were associated with the increased risk of two or more miscarriages compared with women without these polymorphisms. In addition, in three meta-analyses, Chen *et al*.[45], Su *et al*.[46] and Li *et al*.[47] suggested that *PAI-1 4G/5G* polymorphism might be associated with RPL development.

Between-study heterogeneity, a multifactorial phoneme in meta-analyses, is a potential problem when interpreting the results[48-50]. In addition to ethnicity and the source of controls, selection of controls, race variation, age, gender, and prevalence of lifestyle factors might also generate the heterogeneity[35,48,49]. In the present meta-analysis, between-study heterogeneity was observed in all polymorphisms, and thus a random-effect model was used for those genetic models.

There was an inevitable publication bias in our meta-analysis because we retrieved only published studies. Publication bias was assessed by funnel plots whose symmetries were further evaluated by Egger’s linear regression tests. We have suggested that for the recessive model, the publication bias might be owing to the limited number of the selected studies. Therefore, the negative results in our meta-analysis are possibly due to the limited number of publications to determine statistical significance.

To the best of our knowledge, there is no earlier study on the analysis of thrombophilia gene polymorphisms in RPL in the Iranian population. However, in interpreting results of this meta-analysis, some limitations should be addressed. First, there was no sufficient number of relevant studies to explore more comprehensive association between the thrombophilic genes and RPL in the Iranian patients. Second, in this meta-analysis, only published studies were searched. It is possible that some important unpublished studies that meet our inclusion criteria were missed and ignored in the literature search. Therefore, inevitable publication bias might be exist, which could eventually help explain the possible existence of publication bias in the recessive model. Third, the Iranian population is mixed of different ethnicities, including Persian, Azeri, Kurdish, Lurs, Gilaki, Balochi, etc. However, we did not conduct subgroup analyses because insufficient data were available from the primary literature search. Moreover, due to limited individual data, a more precise analysis on other covariates, such as age, number of abortions, and environmental factors should be performed. As a result, more studies with large sample sizes in view of these factors are also desired. Finally, due to the lack of the original data, we did not take potential interactions among gene-gene (especially thrombo-philic genes interactions), gene-environment, or even different polymorphism loci of the same gene, which all may affect RPL risk in the population.

In conclusion, based on the available evidence, the current meta-analysis demonstrates that there is a significant association between the *MTHFR C677T*, *MTHFR A1298C*, *Prothrombin G20210A*, *FVL G1691A*, and *PAI-1 4G/5G* polymorphisms and RPL risk in the Iranian population. In addition, the direction of further research with a larger sample size should focus not only on the simple relationship of thrombophilic genes polymorphisms and RPL risk but also on gene-gene and gene-environment interactions.
